# Emerging Approaches in Intravenous Moderate and Deep Sedation

**DOI:** 10.3390/jcm10081735

**Published:** 2021-04-16

**Authors:** Basavana Goudra, Keira P. Mason

**Affiliations:** 1 Department of Anesthesiology and Critical Care Medicine, Hospital of the University of Pennsylvania, Philadelphia, PA 19104, USA; 2 Department of Anesthesiology, Children’s Hospital Boston, Harvard Medical School, Boston, MA 02115, USA; keira.mason@childrens.harvard.edu

**Keywords:** midazolam, remimazolam, remifentanil, oliceridine

## Abstract

Successful pharmacological innovations that have made a difference in daily practice are rare in the world of anesthesia and sedation. After many years of research, it seems that we finally have two new drug innovations that are likely to change the paradigm of moderate and deep sedation. These are oliceridine and remimazolam. Both have been in development for over a decade. Oliceridine was synthesized in a lab as an entirely new molecule. It is a biased μ- receptor agonist that acts preferentially on the G-protein pathway (which is responsible for analgesia). At least in lower doses, it has minimal effect on the beta-arrestin pathway, which is responsible for unwanted effects of μ-opioid receptor activation such as respiratory depression and gastrointestinal dysfunction. Like any other μ- receptor agonist, it produces appropriate dose-dependent analgesia. Remimazolam is structurally similar to midazolam; however, it has an additional ester linkage that delivers the kinetics of remifentanil. As a result, while pharmacodynamically identical to midazolam, remimazolam is metabolized by ester hydrolysis and subsequently its elimination is rapid and predictable. The present review discusses the two drugs in detail with a particular emphasis on their potential role in moderate and deep sedation.

## 1. Introduction

Benzodiazepines and short-acting opioids have formed the mainstay of moderate sedation, while propofol is considered the sine qua non for deep sedation. As defined by the American Society of Anesthesiologists (ASA), moderate sedation is a drug-induced depression of consciousness during which patients respond purposefully to verbal commands, either alone or accompanied by light tactile stimulation. These patients might still exhibit reflex withdrawal to a painful stimulus and it is not considered a purposeful response. No interventions are required to maintain a patent airway, and spontaneous ventilation is adequate. Cardiovascular function is usually maintained. Patient might exhibit purposeful response (reflex withdrawal from a painful stimulus is not considered a purposeful response) to verbal or tactile stimulation. Indications for monitored anesthesia care include “the need for deeper levels of analgesia and sedation than can be provided by moderate sedation (including potential conversion to a general or regional anesthetic)” [1]. ASA defines deep sedation as a drug-induced depression of consciousness during which patients cannot be easily aroused but respond purposefully following repeated or painful stimulation. The ability to independently maintain ventilatory function may be impaired. Patients may require assistance in maintaining a patent airway, and spontaneous ventilation may be inadequate. Cardiovascular function is usually maintained. In the USA, moderate sedation is largely administered by nurses under the supervision of the physician performing the procedures, whereas propofol is principally administered by certified nurse anesthetists (often under the supervision of an attending anesthesiologist). Moreover, as per the product insert, propofol can only be administered by those with training in airway management.

Variability in the pharmacokinetics (PK) and pharmacodynamics (PD) is a significant limitation for most intravenous anesthetics and sedatives [2,3,4]. Although both PK and PD variability is seen across the age spectrum, the elderly are especially susceptible to sedatives largely because of PD variability. In addition to variability, absence of a reversal agent is a major impediment for wider use of propofol, especially by those without sufficient training in airway control.

Efforts are being made to address the variabilities and other shortcomings in relation to moderate and deep sedation. An early effort to address the FDA requirement of the need for airway trained personnel was the rush to get Fospropofol approved for use by non-anesthesia providers. Fospropofol is a prodrug of propofol with very similar pharmacokinetic and pharmacodynamic properties. Nevertheless, it was not approved for such use due its propensity to cause apnea and airway obstruction similar to propofol. The second major effort was the invention of Sedasys^®^, a device created for self-administration of propofol in an effort to overcome the PK/PD variability. However, for unknown reasons, the manufacturers decided to pull out the device soon after introduction [5,6,7,8,9].

Recently, remimazolam, a newer short-acting benzodiazepine, is being explored as a possible sedative. Although it is mainly studied for gastrointestinal (GI) endoscopic and bronchoscopic procedures, its unique pharmacokinetic profile can make it attractive for any procedure requiring moderate sedation. In addition, it may be used as a potential replacement or adjuvant in general anesthesia. Oliceridine is another new drug invention that is a short-acting injectable opioid which selectively activates a subtype of mu receptor. It is relatively devoid of respiratory depressant properties and might suitably complement benzodiazepines and intravenous anesthetics. Both were recently approved by the Food and Drug Administration (FDA). In the succeeding paragraphs, the authors discuss the utility of remimazolam and oliceridine in the context of moderate and deep sedation.

## 2. Remimazolam (CNS7056)

Remimazolam is an ester-modified benzodiazepine analog [10] that is metabolized by plasma tissue esterases to inactive carboxylic acid metabolites [11]. Remimazolam first received approval in Japan as an intravenous anesthetic in January 2020. South Korea, Japan, China, and the United States are currently the only countries with approved usage as detailed in Table 1. (Table 1) Remimazolam is designed to be an ultra-short-acting anesthetic, metabolized by tissue esterases to inactive metabolites and carboxylic acid, eliminated by first order pharmacokinetics. 

### 2.1. Studies for Approval: Phase 1–3

Phase 1 trials were first reported in 2012. The first phase 1 trial compared a single 2 min IV infusion of remimazolam (0.01–0.35 mg/kg), midazolam (0.075 mg/kg), or placebo. Remimazolam demonstrated a linear clearance approximately 3 times faster than midazolam and independent of body weight. At equi-effective doses of remimazolam and midazolam, median recovery time was 30 min faster with remimazolam [12]. The phase 1 single ascending dose study comparing remimazolam (0.01–0.3 mg/kg) to midazolam (0.075 mg/kg) and placebo reported that there was good predictability of BIS scores, Modified Observer’s Assessment of Alertness/Sedation (MOAA/S) and context sensitive half time for remimazolam, even with prolonged infusions [12]. Maximum effect of remimazolam is achieved within 3 min, unaffected by body weight (65–90 kg studied) [12].

Phase 2a trials were randomized, double blind studies comparing a single dose of remimazolam (0.1–0.2 mg/kg) to midazolam (0.075 mg/kg) for upper gastrointestinal endoscopy. At higher doses, remimazolam achieved adequate sedation for completion of the procedure in 64% of the volunteers, higher than the 44% in the midazolam group [13]. The Phase 2b/3 trials in Japan compared remimazolam (6 or 12 mg/kg/hr) to propofol (2–2.5 mg/kg), both administered until loss of consciousness and then maintained with a titrated continuous infusion. Remimazolam was non-inferior to propofol with respect to efficacy, with remimazolam demonstrating longer time to achieve loss of consciousness and extubation [14].

A multicenter, randomized non-inferiority phase 3 trial performed at 17 centers in China demonstrated non-inferiority of propofol to remimazolam for successful completion of upper gastrointestinal endoscopy. There was a significant decreased incidence of hypotension and respiratory depression with remimazolam and a faster time to achieve recovery [15].

A phase 3 trial in the United States compared the efficacy and safety of remimazolam to midazolam and placebo for colonoscopy at 12 test sites. Primary endpoints reflected completion of colonoscopy, need for rescue medication, and number of repeat doses required in a 15 min interval. Remimazolam was superior to midazolam, achieving the desired endpoints in 91.3 vs 25.2% of patients respectively. Remimazolam achieved faster time to discharge and to return of neuropsychiatric function [16].

### 2.2. Pharmacokinetics and Pharmacodynamics

#### 2.2.1. Intravenous

The PK and PD of a remimazolam infusion was evaluated in healthy adult volunteers. On average, the volunteers lost consciousness after receiving 5 mg/min for 5 min. Maintained on a subsequent 3 mg/min then 1 mg/min for 15 min each, the volunteers were fully alert at an average of 19 min after the final 1 mg/min infusion. Remimazolam demonstrated a multicompartmental pharmacokinetic model with small volume of distribution, rapid clearance, and minimal interindividual variability. The predicted context-sensitive halftime after a 4 h infusion was a mean of 6.8 min [17]. EEG activity during this remimazolam dosing demonstrated an initial increase in beta frequency followed by a late increase in delta. The EEG response to remimazolam does not lend itself to Narcotrend predictability. Following beta activity, however, may be a suitable means to predict depth of sedation and Modified Observer’s Assessment of Alertness and Sedation scores [17].

A two-part study performed on healthy volunteers in China demonstrated that a single ascending dose of remimazolam (0.025-0.4 mg/kg) has a half-life which ranges from 34 to 59 min. Peak sedation effect was observed within 1–2 min at does greater that 0.075 mg/kg. With a 2 h continuous infusion of remimazolam, recovery was achieved faster and sedation depth was deeper as compared to volunteers who received a continuous infusion of midazolam [18].

#### 2.2.2. Intranasal

A randomized, double-blind, controlled clinical trial compared the PK, PD, and safety of intranasal remimazolam (10, 20, and 40 mg) with 4 mg IV remimazolam or placebo. The intranasal dose was administered as a powder or solution. At higher intranasal (IN) doses, the bioavailability decreased, likely from incomplete delivery and resultant oral administration. Although bioavailability was approximately 50%, clinical effects were consistent and timely with a Tmax of 10 min; the clinical applicability of this intranasal formulation is non-existent, given the extreme pain on administration [19]. New formulations will be necessary in order to reduce the pain on administration for IN to be an offered route.

#### 2.2.3. Oral

The bioavailability of remimazolam by the oral route is very poor. A study in healthy adults reported that even at doses of 480 mg, there were no clinically significant pharmacodynamic effects. Even in the presence of alcohol, although there was a dose-dependent increase in remimazolam (maximum observed plasma concentration), the clinical effects were mild. The bioavailability of oral remimazolam was reported at 1.2–2.2%, eliminating its use by the oral route [20].

### 2.3. Clinical Reports

Small case reports have described the successful use of remimazolam and remifentanil for spine surgery with intraoperative motor-evoked potentials [14,15,16,17,18,19,20,21]. It has demonstrated success in a prospective, double-blind, multicenter (30 sites in United States), parallel group trial comparing it to midazolam and placebo for bronchoscopy in adults. Remimazolam (5 mg initial and 2.5 mg supplement as needed) achieved greater success than midazolam (1–1.75 mg initial and 0.5–1 mg supplement as needed) at 80.6% and 32.9% respectively, with a median of 6 min faster time to wake-up [22].

The exact dosing for subpopulations, taking into account covariates, has not been studied in large clinical trials. Particularly in ASA ≥ 3 and the elderly, it is important to clarify dosing. A pooled analysis of 4 clinical trials of the induction and maintenance of general anesthesia with remimazolam was designed to evaluate effect of covariates on time to loss of consciousness, time to offset (termination of infusion to extubation), and the steady-state infusion rate necessary to achieve adequate sedation as determined by BIS/Narcotrend. Time-to-event and factors affecting steady-state infusion rate were analyzed using Kaplan–Meier plots and linear regression analysis, respectively. Induction dose, age, body mass index (BMI), and timing of opiate delivery were significant covariates for time to loss-of-consciousness. ASA status did not affect time to loss-of-consciousness. The obese and elderly had slightly faster time to loss of consciousness (12 and 20 s respectively). Time to extubation was determined by infusion rate and depth of sedation, unaffected by BMI, age, and ASA class. The clinical significance of these factors, albeit statistically significant, in reality are clinically of minimal significance as providers will likely decrease dosing in the obese and titrate down dosing in anticipation of extubation [16,17,18,19,20,21,22,23].

A double blind, randomized, multi-center (3 sites in United States), parallel group trial was designed to compare remimazolam to open-label midazolam for ASA 3 and 4 adults undergoing colonoscopy. Remimazolam achieved greater in procedural success than midazolam, 87.1% versus 13.3% respectively. There was no difference in adverse events [17,18,19,20,21,22,23,24].

The psychomotor effects of remimazolam were evaluated and compared to midazolam in a study performed in China. After a 2 h continuous infusion of remimazolam, with depth of anesthesia maintained at a BIS 40–60, time to full alertness was accelerated by 3.5 min in response to flumazenil. Flumazenil demonstrated a return to psychomotor function in the immediate recovery period. At 2 h post infusion, however, a delayed recall was exhibited, a deficiency that was not corrected by flumazenil.

## 3. Oliceridine

Oliceridine belongs to a class of opioids that target G protein-coupled receptors to produce its beneficial effects. μ-opioid receptor (just like other opioid receptors) is a G protein-coupled receptor. Opioids such as morphine bind to μ-opioid receptors and activate 2 downstream signaling pathways. The first is the G-protein pathway which is linked to analgesia, and the second is β-arrestin recruitment which is responsible for unwanted effects of μ-opioid receptor activation. The β-arrestin activation is the main factor which limits the efficacy of opioids [25]. On binding to the µ-opioid receptor, oliceridine selectively activates guanosine triphosphates and this action is responsible for analgesia. The second pathway results from activation of β-arrestin which is responsible for many adverse effects such as respiratory depression, GI side effects, hyperalgesia, tolerance, and addiction which is relatively spared by these G-protein biased opioid agonists [25]. In other words, it differentially activates G-protein coupling while mitigating β-arrestin recruitment. However, the selectivity is not absolute and is less obvious in higher doses. By using a tail flick assay in mice, Liang et al. demonstrated that oliceridine has a 4-fold more potent analgesic activity than morphine (*p* < 0.001). In addition, it caused less tolerance (*p* < 0.001) and opioid-induced hyperalgesia than morphine after 4 days of ascending-dose administration [26].

On 8 August 2020, FDA gave approval to Oliceridine (Olinvyk^®^ Trevena, PA, USA). Similar to many novel drugs that enter the market, Trevena conducted many phase 1, 2, and 3 studies and presented the data to the FDA and its committee responsible for providing advice and guidance on new drugs producing anesthesia and analgesia. As per the data presented at the Advisory Committee meeting on 11 October 2019, oliceridine is shown to exhibit dose-dependent reductions in pain scores. In terms of potency, it is approximately five times more potent than morphine. More importantly, it exhibits a wider therapeutic window. The onset of action to produce effective and analgesia is 3–4 min.

### Approved and Potential Indications

Presently, the only FDA approved indication for Olinvyk is for patient-controlled analgesia (PCA), IV infusion, or bolus in post anesthesia care unit and post-operative phase for pain relief. The recommended initial dose is 1.5 mg that can be administered by a healthcare provider followed by access to patient demand doses with a 6-min lock-out. A demand dose of 0.35 mg is recommended that may be increased to 0.5 mg in selected patients. Healthcare workers are allowed to administer 0.75 mg beginning 1 h after the initial dose, and hourly thereafter as needed. Pain relief is experienced in 2–5 min, similar to fentanyl. Many touted benefits such as reduced respiratory depression and less dependence and abuse liability are mainly demonstrated at lower doses [27].

Although there are no studies demonstrating its potential role in moderate and deep sedation, the authors expect oliceridine to play a major role in this area. Some of the purported shortcomings that could be detrimental when used as an infusion or multiple doses are less of an issue when employed for short duration procedural sedation. Such drawbacks stem from a lack of demonstrable selectivity at higher doses and include addiction liability, hyperalgesia, GI side effects, and respiratory depression. They will continue to limit the amount of Olinvyk^®^ we can administer. However, during procedural sedation, the dose required is significantly less than for general anesthesia and post-operative pain. As a result, the supposed benefits are likely to be real. 

Administration of midazolam and fentanyl (occasionally with diphenhydramine) is a popular technique for moderate sedation. However, unexpected hypoxemia (SpO_2_ ≤ 90%) is frequently reported with moderate sedation, both during the procedure and in the post-procedure period. Often hypoxemia occurs in patients without serious co-morbid illness and with low body mass index [28]. Desaturation with moderate sedation is especially likely in those of the age of 60 years old or more, and ASA III score [29].

In addition, deaths from opioid abuse have become increasingly common especially in the USA. In the USA, in 2017, over 47,000 deaths were recorded due to prescription and nonprescription opioid overdose, and deaths are projected to reach nearly 82,000 by 2025 [30]. Concerns have been raised about the dangers of introducing opioids perioperatively to an opioid naive patient. One study in 2016 found that male and elderly patients (over 50 years of age) with histories of substance abuse had a greater risk of becoming opioid dependent post-surgery where opioids were introduced [31].

Anesthetics such as propofol and short acting opioids such as remifentanil are also routinely administered intravenously using a target-controlled infusion (TCI) system. Pharmacokinetically speaking, both remimazolam and oliceridine are appropriate for TCI. The context-sensitive decrement times (i.e., the times for a defined decrease in plasma and effect site concentrations such as by 25%, 50%, and 75% after continuous target controlled infusion of variable length) are calculated for remimazolam [17,18,19,20,21,22,23,24,25,26,27,28,29,30,31,32]. After an infusion of 4 h, the plasma context-sensitive halftime of remimazolam was 6.8 ± 2.4 min, while for the effect site concentration, it was 12 ± 2 min. This is much shorter than midazolam (122 min after an infusion of 8 h). Similar data are not yet available for oliceridine. 

Along with procedural sedation, remimazolam manufacturers are also evaluating its use for induction and maintenance of general anesthesia, and ICU sedation [33]. However, it might have drawbacks similar to any benzodiazepine such as delirium, although short context sensitive halftime might offer some protection [14]. There are no studies that have addressed the potential role of oliceridine for ICU sedation.

## 4. Conclusions

Both remimazolam and oliceridine have a definite role in the provision of intravenous moderate and deep sedation. They may be administered as adjuvants to more established sedative/anesthetics such as propofol, etomidate, ketamine, and dexmedetomidine; although more studies are necessary to understand all the pharmacokinetic and pharmacodynamic interactions. Administration of remimazolam and remifentanil together to produce moderate sedation, thereby exploiting the benefits of both and limiting their possible individual adverse effects, has its appeal. Once again, more scientific literature is needed.

## Figures and Tables

**Table 1:** Approvals of Remimazolam.

Country	Date of Approval	Indication	Marketing Authorization Holder
Japan	23 January 2020	general anaesthesia	Mundipharma
USA	2 July 2020	procedural sedation	Acacia Pharma
China	20 July 2020	procedural sedation	Yichang Humanwell
South Korea	7 January 2021	general anaesthesia	Hana Pharm
